# MSIpred: a python package for tumor microsatellite instability classification from tumor mutation annotation data using a support vector machine

**DOI:** 10.1038/s41598-018-35682-z

**Published:** 2018-12-03

**Authors:** Chen Wang, Chun Liang

**Affiliations:** 1 Department of Biology, Miami University, Oxford, OH 45056 USA; 2 Department of Computer Science & Software Engineering, Miami University, Oxford, OH 45056 USA

## Abstract

Microsatellite instability (MSI) is characterized by high degree of polymorphism in microsatellite lengths due to deficiency in mismatch repair (MMR) system. MSI is associated with several tumor types and its status can be considered as an important indicator for patient prognosis. Conventional clinical diagnosis of MSI examines PCR products of a panel of microsatellite markers using electrophoresis (MSI-PCR), which is laborious, costly, and time consuming. We developed MSIpred, a python package for automatic MSI classification using a machine learning technology – support vector machine (SVM). MSIpred computes 22 features characterizing tumor somatic mutational load from mutation data in mutation annotation format (MAF) generated from paired tumor-normal exome sequencing data, subsequently using these features to predict tumor MSI status with a SVM classifier trained by MAF data of 1074 tumors belonging to four types. Evaluation of MSIpred on an independent testing set, MAF data of another 358 tumors, achieved overall accuracy of ≥98% and area under receiver operating characteristic (ROC) curve of 0.967. Further analysis on discrepant cases revealed that discrepancies were partially due to misclassification of MSI-PCR. Additional testing of MSIpred on non-TCGA data also validated its good classification performance. These results indicated that MSIpred is a robust pan-tumor MSI classification tool and can serve as a complementary diagnostic to MSI-PCR in MSI diagnosis.

## Introduction

Microsatellites are tandemly repeated sequences with typical repeat unit length varying from 1 to 6 bases^1^. Slippage events during DNA replication can lead to gain or loss of repeat units from microsatellite loci throughout genome. Under normal circumstances, these spontaneous mutations can be sensed and corrected by mismatch repair (MMR) system. When MMR system is inactivated or affected due to mutations or epigenetic silencing in its associated genes, including *MSH2*, *MSH3*, *MSH6*, *MLH1*, *MLH3*, *PMS1*, *PMS2*^2^, consequent high degree of polymorphism in microsatellite length, defined as microsatellite instability (MSI), frequently occurs^3^. Clinical diagnosis of MSI is usually achieved by examining lengths of PCR products of five informative microsatellite loci (BAT25, BAT26, D2S123, D5S346, and D17S250) of National Cancer Institute microsatellite panel by capillary electrophoresis (known as MSI-PCR method). According to Bethesda guideline^4^, tumors are termed as microsatellite stable (MSS) if none of five loci tested is mutated, and tumors are termed as microsatellite instability low (MSI-L) if only one tested locus is mutated and microsatellite instability high (MSI-H) if two or more tested loci are mutated. MSI is best known in colorectal tumor where clear clinical implications have been established. 15% cases of colorectal tumors are observed with MSI. 3% of them are inheritable and are defined as Lynch syndrome where inactivating germline mutations in genes of MMR system are detected. The other 12% are sporadic cases caused by somatic mutations in genes of MMR system^5,6^. MSI-H colorectal tumors tend to have better prognosis and are less prone to metastasis^7^. Though less understood, MSI is also reported in endometrial, gastric, glioblastoma and prostate tumors^8,9^. Classification of MSI is of great importance due to its significant relationship with therapeutic decisions, but clinical diagnosis of MSI by MSI-PCR is laborious, costly, and time consuming. With the development of next generation sequencing (NGS) technology, several computational tools utilizing NGS data were developed for MSI diagnosis. MSIsensor^10^ and mSINGS^11^ determine tumor MSI status by measuring prevalence of unstable microsatellite loci in paired tumor-normal sequencing data. These two tools showed good performance but they both require lots of computational resources since they directly examine aligned reads in BAM format. MSIseq, MOSAIC, and MIRMMR^12–14^ utilize machine learning algorithms to predict MSI status. MSIseq and MOSAIC implemented decision tree classifiers for MSI classification depending on just a single feature derived from tumor somatic mutation information, which is prone to over-fit their training datasets. MIRMMR built a logistic regression classifier requiring both methylation and mutation information of genes belonging to MMR system. A recent study revealed that, in addition to MSI, MMR-deficient tumors also display relatively higher somatic mutational load^15^. Thus, it is a reasonable direction to predict tumor MSI status through tumor mutation information obtained by exome sequencing. Recently, mutation calling has become a routine analysis of paired tumor-normal sequencing data. Several different pipelines for mutation calling have been published^16–21^. These pipelines typically create a file in mutation annotation format (MAF). MAF files provide somatic mutation information of tumors including single nucleotide mutations, micro-indels, and their detailed translational/regulatory effects (for example, missense, nonsense, and silence mutations) on annotated genes. In this study, we explored distributions of somatic mutational load in MSI-PCR termed MSI-H and non MSI-H (MSS and MSI-L) tumors using their corresponding MAF files obtained from The Cancer Genome Atlas (TCGA) Research Network^22^, and subsequently developed a python package, MSIpred, implementing a pan-tumor binary MSI classifier to predict MSI status from tumor MAF files.

## Results

### Distributions of somatic mutational load in MSS and MSI-H tumors

**Figure 1:**
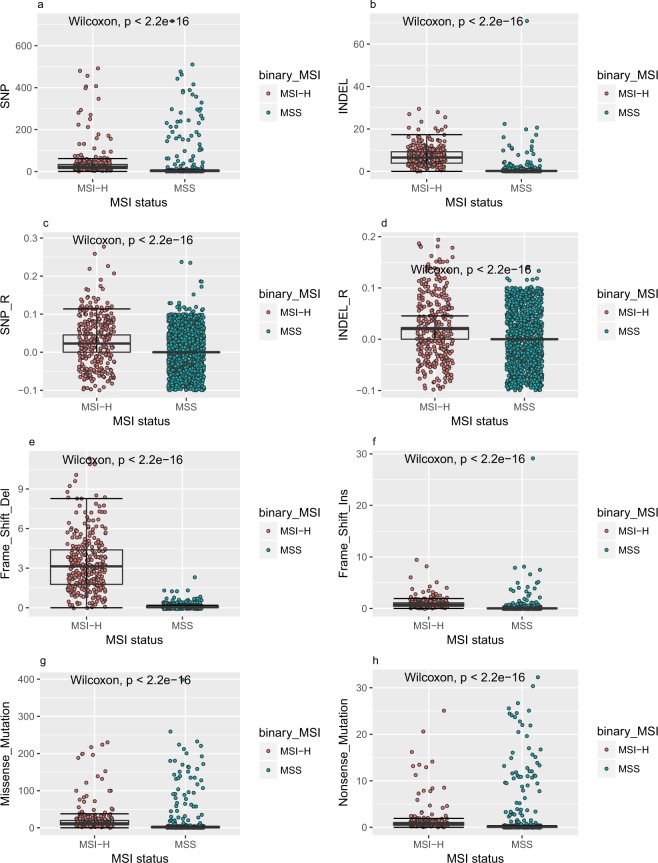
MSS and MSI-H tumors possessed different somatic mutational load. Boxplots for eight somatic mutational load features of MSS (a combination of MSI-L and MSS) tumors and MSI-H tumors were given. X axis of each subplot denotes two MSI status and Y axis denotes counts (per megabase (Mb)) of a mutational load feature. P-values obtained by Wilcoxon rank sum test were labeled on each subplot. From (**a**–**h**), each subplot denotes following features: SNP, INDEL, SNP_R, INDEL_R, Frame_Shift_Del, Frame_Shift_Ins, Missense_Mutation, and Nonsense_Mutation, respectively.

We calculated 22 features characterizing somatic mutational load of 1432 tumors of four types (see Table 1, COAD: colon adenocarcinoma, READ: rectal adenocarcinoma, STAD: stomach adenocarcinoma, and UCEC: uterine corpus endometrial carcinoma) using mutation data from tumor MAF files. Of all 22 features, 1–9, previously used by MSIseq^12^, describe a general type (single nucleotide variants or micro-indels) of a mutation. 10–22 describe detailed classification of a mutation based on its translational and/or regulatory effects, which can provide critical information about how deleterious a mutation is (see Supplementary Table S1). All of these 22 features displayed skewed distributions in total 1432 tumors (see Supplementary Fig. S1). As a recent study revealed that MSI-L tumors were consistent with MSS tumors in terms of their MSI burden^13^, we combined MSS and MSI-L tumors together, and denoted them as MSS in this study. Referring to tumor clinical data provided by TCGA, 1123 of 1432 tumors were determined as MSS using MSI-PCR, the other 309 tumors were determined as MSI-H (see Table 2). MSI-H tumors displayed significantly higher counts of mutations (both single nucleotide variants and micro-indels) per megabase than MSS tumors throughout exome sequences and in simple sequence repeat regions. MSI-H tumors also tended to possess more deleterious mutations, such as mutations causing shifts in open reading frames, missense mutations, and nonsense mutations (Fig. 1). By pairing these 22 features, a clear separation between MSS tumors and MSI-H tumors can be observed (see Supplementary Fig. S2). Wilcoxon rank-sum tests of each of these 22 features between MSS and MSI-H tumors were performed, and results indicated that all these 22 features were differently distributed between MSS and MSI-H tumors.

**Table 1:** Composition of training and testing data sets according to tumor types.

Project	Training	Testing
COAD	278	99
READ	92	39
STAD	334	103
UCEC	370	117
Total	1074	358

**Table 2:** Composition of training and testing data sets according to MSI-PCR determined MSI status.

MSI status	Training	Testing
MSS*	705	227
MSI-L*	138	53
MSI-H	231	78
Total	1074	358

*Both MSS and MSI-L are labelled as MSS for binary classification in our study.

### Analysis of 22 features used for mutational load characterization

1432 tumors were then randomly split into two datasets, training and testing, using 3:1 ratio within each tumor type. Accordingly, the training set incorporated 1074 tumors of all four types, and the testing set incorporated 358 tumors of four types. Detailed information about these two datasets were presented in Tables 1 and 2. In order to gain some insights about the contributions of these features to MSI classification, we first trained a random forest classifier using all 22 features of 1074 tumors belonging to the training set. The importance scores of all these 22 features were then obtained (see Supplementary Table S2) from the random forest classifier to evaluate contributions of these features^23^. According to their importance scores, those 13 new features other than the 9 features selected by MSIseq^12^ greatly impact the classification results, especially Frame_Shift_Del and Frame_Shift_Ins. Besides, all 22 features contributed to the classification of MSS and MSI-H tumors in terms of their importance scores obtained from the random forest classifier. Thus, we decided to keep all 22 features for further implementation.

### Classification of MSI using Support Vector Machine

We then developed a pan-tumor support vector machine (SVM) classifier with a radial basis function kernel for MSI classification using aforementioned 22 features of all tumors from the training set. After the SVM classifier was trained, we released a python package called MSIpred (Microsatellite Instability Predictor) implementing that SVM classifier and a bioinformatics pipeline to compute those required features from MAF files to perform MSI auto classification.

### MSIpred is accurate for MSI classification

**Figure 2:**
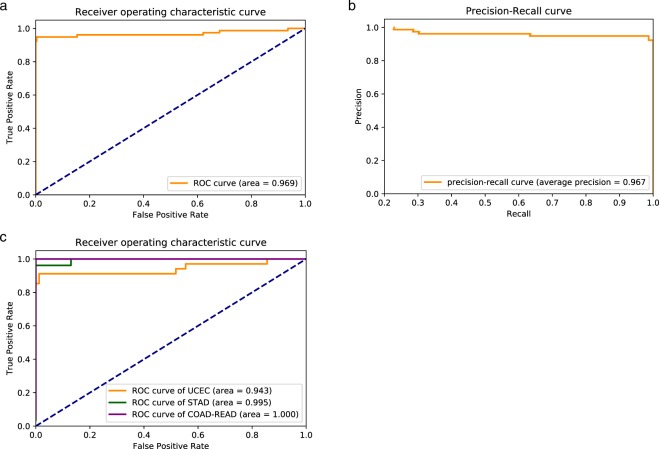
Performance of MSIpred. (**a**) Receiver operating characteristic (ROC) curve of MSIpred. The blue dash line denotes a line with slope of 1 and intercept of 0. (**b**) Precision-recall curve of MSIpred. Both ROC curve and precision-recall curve were obtained by applying MSIpred on the 358-tumor testing set. (**c**) ROC curves of MSIpred for different tumor types. Endometrial tumors (UCEC): orange, 117 tumors; stomach tumors (STAD): green, 103 tumors; colorectal tumors (COAD-READ): purple, 138 tumors.

We applied MSIpred to that 358-tumor testing set and evaluated its performance by finding concordances between MSIpred predicted MSI status and MSI-PCR determined MSI status provided by TCGA, using several different metrics. In this study, we denoted MSS tumors as negative samples while MSI-H tumors as positive ones. MSIpred finally achieved a sensitivity (recall) of 0.936, specificity of 0.996, precision of 0.986, G mean of 0.966, F1 score of 0.961, and overall accuracy of 0.983. ROC curve (Fig. 2a) of MSIpred achieved area under curve of 0.969. Precision-recall curve (Fig. 2b) of MSIpred achieved average precision of 0.967. These results indicated that MSIpred is a robust tool for MSI classification, and revealed that MSS tumors and MSI-H tumors possessed distinct somatic mutational loads.

### MSIpred is robust across different tumor types

MSIpred was designed to perform pan-tumor MSI classification since it didn’t incorporate tumor type as a feature when predicting MSI status. It is reasonable to evaluate performance of MSIpred on each tumor type individually since different types of tumors may hold different signatures of somatic mutational load. Hence, we then evaluated performance of MSIpred on each tumor type individually using the 358-tumor (UCEC: 117 tumors; STAD 103 tumors; COAD and READ :138 tumors) testing set. ROC curves for endometrial, stomach, and colorectal tumors were plotted (Fig. 2c). For colorectal tumors and stomach tumors, MSIpred achieved area under curve of 1.0 and 0.995, respectively. Area under curve for endometrial tumor is slightly lower with a value of 0.943. These results indicated that MSIpred didn’t over-fit a particular tumor type and MSIpred was robust and generic on different tumor types.

### Analysis of discrepant cases

**Figure 3:**
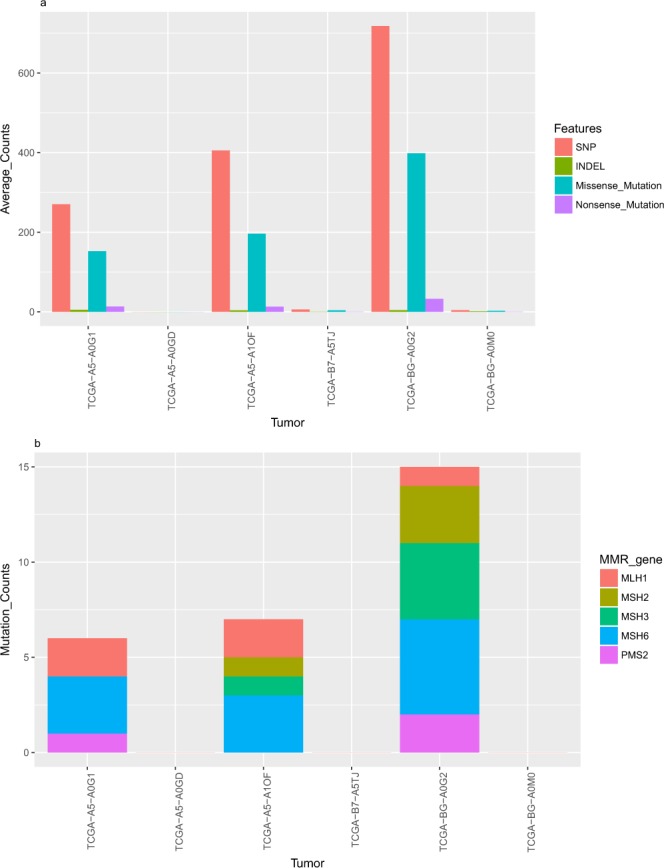
Analysis of discrepant cases. (**a**) Bar plot of four features (SNP, INDEL, Missense_Mutation, Nonsense_Mutations) characterizing somatic mutational load of six discrepant tumors (**b**) Mutation profiles of six discrepant tumors in terms of genes in MMR system.

We further investigated somatic mutational load (characterized by aforementioned 22 features) for those tumors whose MSIpred predicted MSI status and MSI-PCR determined MSI status were discrepant. In the 358-tumor testing set, 5 (4 endometrial, 1 stomach) of 78 MSI-PCR determined MSI-H tumors were classified as MSS by MSIpred (false negative). 1 (endometrial) of 280 MSI-PCR determined MSS tumors was classified as MSI-H (false positive) by MSIpred. As shown in Fig. 3a, three (TCGA-B7-A5TJ, TCGA-A5-A0GD, TCGA-BG-A0M0) of five false negative tumors possessed quite low somatic mutational load, indicating their MMR system might still be intact. The other two false negative tumors possessed high somatic mutational load. The only false positive tumor (TCGA-BG-A0G2, see Fig. 3a) also possessed relatively high somatic mutational load, indicating that its MMR system might have been inactivated or affected. We then analyzed their corresponding MAF files with special foci on five genes involved in MMR system including *MLH1*, *MSH2*, *MSH3*, *MSH6* and *PMS2*. For those three false negative tumors (TCGA-B7-A5TJ, TCGA-A5-A0GD, TCGA-BG-A0M0) with low somatic mutational load, no mutation was found in these five MMR genes. For the other two false negative tumors with high somatic mutation load, mutations were detected in MMR genes: TCGA-A5-A1OF possessed mutations in four (*MLH1*, *MSH2*, *MSH3*, *MSH6*) of five MMR genes; TCGA-A5-A0G1 possessed mutations in three (*MLH1*, *MSH6*, *PMS2*) of five MMR genes. The only false positive tumor, TCGA-BG-A0G2, possessed mutations in all five MMR genes (Fig. 3b). This result suggests that the MSI-PCR results might not be very accurate for these cases.

### Validation of MSIpred using non-TCGA MAF data

As both of the training set and testing set data were obtained from TCGA, the performance of MSIpred on the aforementioned testing set might be overoptimistic. We further decided to validate the performance of MSIpred with another 390-tumor testing set whose data were generated by non-TCGA projects. This 390-tumor testing set consisted of MAF files of 368 colorectal tumors^24^ and 22 stomach tumors^25^. These MAF files and their corresponding binary (cBioPortal directly provided binary MSI status) MSI status determined by MSI-PCR were all obtained from cBioPortal^26,27^. 90 of 390 tumors were determined as MSI-H and the rest 300 were determined as MSS by MSI-PCR. With this non-TCGA testing set, MSIpred achieved a sensitivity (recall) of 0.778, specificity of 0.990, precision of 0.959, G mean of 0.877, F1 score of 0.859, and overall accuracy of 0.941. Compared to the performance of MSIpred on the previous 358-tumor TCGA testing data, the sensitivity of MSIpred on non-TCGA testing data was lower, but still reached a good overall accuracy.

### Comparison of MSIpred with MSIseq

Among previously published software tools, MSIseq^12^ is the only one that also predicts MSI status solely from tumor MAF files. Our tool MSIpred also utilized nine mutation features that were first proposed by MSIseq. Therefore, we compared performances of MSIseq with MSIpred in this study. The 390-tumor non-TCGA testing set that was utilized to validate performance of MSIpred was also used to evaluate MSIseq with its default decision tree classifier. MSIseq gave overall accuracy of 0.918, sensitivity of 0.667, precision of 0.968 and specificity of 0.993 (see Table 3). These results revealed that MSIseq was good at recognizing negative samples (MSS) but poor at recognizing positive samples (MSI-H). Thus, MSIpred achieved higher sensitivity and accuracy than MSIseq.

**Table 3:** Performances of original MSIpred, modified MSIpred, and MSIseq on 390-tumor non-TCGA testing set.

Tools	non-TCGA testing set size							
	MSS	MSI-H	*Se* (*recall*)	*Sp*	*Pre*	*G mean*	F1	ACC
MSIpred (22 features)	300	90	0.778	0.990	0.959	0.877	0.859	0.941
MSIpred (9 features)			0.622	0.997	0.982	0.787	0.762	0.910
MSIseq			0.667	0.993	0.968	0.814	0.789	0.918

### 13 new features improved performance of MSIpred

Of all 22 features utilized by MSIpred, 13 (see Supplementary Table S1, row 10–22) were new features for MSI classification. These 13 features characterized detailed translational or regulatory effects of mutations. We validated the positive impacts of these 13 new features on MSI classification by comparing performances of two different versions of MSIpred on the aforementioned 390-tumor non-TCGA testing set: the original MSIpred that utilized all 22 (9 inherited from MSIseq plus 13 new features) features and a modified MSIpred that only utilized 9 features proposed by MSIseq (see Supplementary Table S1, row 1–9). Results showed that by adding 13 new features, performance of MSIpred was improved (see Table 3). The sensitivity of MSIpred was improved from 0.667 to 0.778 and overall accuracy was improved from 0.910 to 0.941.

## Discussion

We utilized 22 features, which can be computed from tumor MAF files, to characterize somatic mutational load of tumors by considering both mutation types and their detailed translational/regulatory effects. All these 22 features were differentially distributed in MSI-PCR determined MSS and MSI-H tumors, indicating that these features could serve as useful predictors for MSI classification. Further analysis on these 22 features using a random forest classifier revealed that all these 22 features contributed to the classification of MSS and MSI-H tumors. We then developed a python package called MSIpred, which implemented a SVM classifier trained by those 22 features of a 1074-tumor training set, and a bioinformatics pipeline to compute those required features from MAF files to perform automatic binary MSI classification. If more data is available for training, MSIpred allows redo of the training step in order to get a better classifier. Those MAF files that MSIpred utilized as inputs were generated from paired tumor-normal exome sequencing data. An evaluation of MSIpred by finding concordances with MSI-PCR determined MSI status on a 358-tumor testing set showed that MSIpred is a robust pan-tumor MSI classification tool with high accuracy, sensitivity, and specificity.

Using MSI-PCR determined MSI status as the experimental reference, 6 discrepancies including five false negative cases and one false positive case were observed in MSIpred classification results. Further analysis of their somatic mutational load and mutation profiles in terms of genes belonging to MMR system revealed that three of five false negative tumors were likely to possess intact MMR systems, while the other two false negatives were likely to possess inactivated or insufficient MMR systems. The only false positive case was also likely to possess an inactivated or insufficient MMR system. These results all together revealed that some of the discrepancies may due to misclassification of MSI-PCR method when considering tumor mutation profiles (see Fig. 3). For those three false negatives with intact MMR systems and the only false positive case, their MSIpred predicted MSI status were more persuasive while for the rest two false negative cases, MSI-PCR termed MSI status were more likely to be correct. Thus MSIpred can serve as a reliable complementary tool for MSI diagnosis beyond the conventional MSI-PCR method. Besides, as MSIpred only requires MAF files for MSI classification, it enables researchers to better utilize NGS data from public domain.

Several computational tools for MSI classification based on NGS data were published. MSIsensor^10^ and mSINGS^11^ both examine aligned reads in “BAM” format, and try to derive MSI status by measuring prevalence of unstable microsatellite loci throughout genome. MOSAIC^13^ is another software that utilizes sequencing data in “BAM” format for MSI classification. However, MOSAIC doesn’t directly compare distributions of unstable microsatellite loci in paired tumor-normal data, instead, it implemented a weighted tree classifier to perform MSI classification using average gain of novel microsatellite alleles. BAM files are often very large and need large amount of computational resources to manipulate. Mutation calling has become a routine analysis for NGS data and yields tumor mutation data in MAF format. MSIseq^12^ and MIRMMR^14^, two MSI classification tools that either solely or partially utilize MAF data for MSI classification have been released. MSIseq implemented a decision tree classifier using features computed from MAF files as well as a feature of tumor types, which would potentially limit its application to some rare tumors. In addition, MSIseq only makes use of general types of mutations (single nucleotide variants or micro-indels) without considering their translational and regulatory effects. Unlike MSIseq, which only requires MAF data, MIRMMR also requires additional tumor methylation data to predict MSI status by a logistic regression classifier. In our study, MSIpred incorporated 9 of 10 features (eliminated tumor type) proposed by MSIseq, and added another 13 features that take potential translational and regulatory effects of mutations into account. By applying MSIpred and MSIseq to another 390-tumor non-TCGA testing set, the good performance of MSIpred was further validated, though a decline in its sensitivity was observed. This underestimation of MSI using non-TCGA data could potentially be explained by that MMR genes can also be repressed by epigenetic mechanisms. Apart from that, MSIpred did not consider individual microsatellite locus when doing classification, but individual microsatellite locus might be informative in terms of MSI classification. Compared to MSIseq, MSIpred still reached a better sensitivity and overall accuracy. A modified MSIpred that only utilized 9 (the features previously utilized by MSIseq) of 22 features was also applied to the same non-TCGA testing set, resulting a lower sensitivity and overall accuracy. It implied the positive informative effects of those 13 new features in MSI classification.

We finally conclude that MSIpred is a robust tool for pan-tumor MSI classification from tumor MAF data, and is available as a python 2 package. We anticipate that MSIpred will have a wide usage in MSI clinical diagnosis.

## Methods

### Input and output

MSIpred requires MAF files containing tumor somatic mutation annotation information derived from paired tumor-normal whole exome sequencing data, a reference file indicating loci of simple sequence repeats regions throughout genome, and length (Mb) of overall captured sequences used for exome sequencing. MSIpred then computes 22 features characterizing somatic mutational loads of tumors with the implemented bioinformatics pipeline. Subsequently, these features are utilized for prediction of MSI status. The final output of MSIpred is a pandas dataframe containing predicted MSI status of tumors.

### Implementation

**Figure 4:**
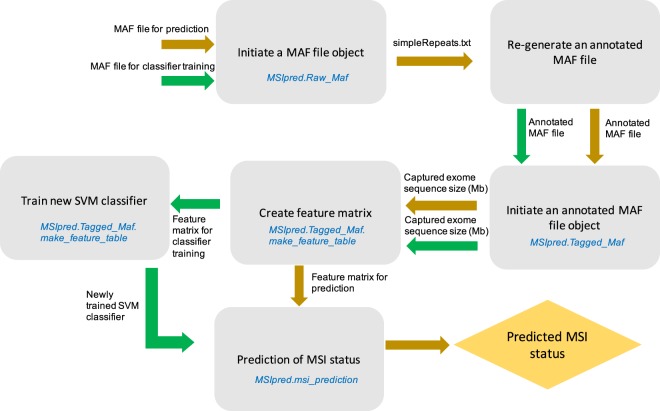
Workflow for MSIpred. MSIpred provides a python class of MAF file (*MSIpred*.*Raw_Maf*), which requires a tumor MAF file to create a MAF file object. After a MAF file object has been created, a method (*Raw_Maf*.*create_tagged_maf*) associated with this object will re-generate an annotated MAF file by adding one extra column called “In_repeats”, which indicates whether mutation events happen in simple repeats region or not, given a reference file indicating loci of simple repeats regions throughout genome (simpleRepeats.txt file). Then, a class of annotated MAF file provided by MSIpred (*MSIpred*.*Tagged_Maf*) takes the aforementioned annotated MAF file to create an object of annotated MAF file. A method (*Tagged_Maf*.*make_feature_table*) associated with annotated MAF file object takes the size (Mb) of captured exome sequences used for exome sequencing to calculate 22 features for somatic mutational load characterization of all tumors embedded in the very first MAF file, and returns a feature matrix as a pandas dataframe. Finally, *MSIpred*.*msi_prediction*, a function provided by MSIpred, takes that feature matrix, and gives the final predicted MSI status for all tumors with the help of a implemented SVM classifier. MSIpred also allows users to train their own SVM classifier by a function called *MSIpred*.*svm_training* using newly obtained MAF data and known MSI status of tumors. The newly trained classifier can be utilized in *MSIpred*.*msi_prediction* function for MSI prediction and classification.

MSIpred is written and tested under python 2 (version 2.7.12) and is freely available as a python package. It requires pandas (version 0.20.3)^28^, intervaltree (version 2.1.0)^29^, and scikit-learn (0.19.1)^30^ packages to work properly. First, a MAF file is annotated by adding an additional column “In_repeats”, indicating whether mutation events took place in simple sequence repeats region or not. The annotated MAF file generated by last step is then utilized to extract 22 features characterizing somatic mutational load for all tumors embedded in this annotated MAF file. These 22 features of all tumors are then combined into a feature matrix as a pandas dataframe. Finally, MSIpred calls the implemented SVM classifier to accurately predict tumor MSI status using the aforementioned feature matrix, and returns results as a pandas dataframe. MSIpred also allows users to train new SVM classifier given new MAF data and known MSI statuses of tumors. Subsequently, the newly trained SVM classifier can be utilized for MSI prediction. A general workflow of MSIpred is presented in Fig. 4.

### Somatic mutation data and clinical MSI status of tumors

MAF files of somatic mutation data created by MuTect mutation calling pipeline were collected from four projects (COAD: colon adenocarcinoma, READ: rectal adenocarcinoma, STAD: stomach adenocarcinoma, and UCEC: uterine corpus endometrial carcinoma) of TCGA^31–33^.

MSI-PCR determined MSI status of all tumors were collected from tumor clinical data provided by TCGA. To retrieve these data from TCGA, a R package called TCGAbiolinks (version 2.5.12)^34^ was used for data query in this study. In total, MAF files together with MSI-PCR determined MSI status of 1432 tumors (COAD: 377, READ: 131, STAD: 437, UCEC: 487) were used in this study.

### Feature selection

We incorporated 9 features (see Supplementary Table S1, row 1–9) proposed by MSIseq^12^. These nine features were calculated based on information of ‘Variant_Type’ columns of MAF files. These nine features take account mutations in simple sequence repeats region specially, thus we need a reference file for loci of simple sequence repeats region in human genome. We retrieved loci of simple sequence repeats for GRCh38 (Genome Reference Consortium Human Reference 38) from a table called “SimpleRepeats” in UCSC genome annotation database (http://hgdownload.cse.ucsc.edu/goldenPath/hg38/database/simpleRepeat.txt.gz). Although, typical unit lengths of microsatellites vary from one to six base pairs, but a recent study pointed out that microsatellite loci with long unit lengths cannot be detected reliably by using 100 bp (which is the reads length utilized by TCGA) sequencing reads^35^. Thus we only retrieved loci of simple sequence repeats whose unit lengths are less than and equal to five base pairs. Besides, we utilized another 13 features (see Supplementary Table S1, row 10–22), which were calculated from information of ‘Variant_Classification’ columns of MAF files. These 13 features gave more detailed information about potential translational and regulatory effects of mutations that can differentiate deleterious mutations from not deleterious ones. In general, all these 22 features denote counts of different kinds of mutations normalized by lengths (Mb) of captured exome sequences. For all 1432 tumors used in this study, their captured exome sequence lengths were obtained from their TCGA project marker papers (COAD: 44 Mb, READ: 44 Mb, STAD: 50 Mb, UCEC: 44 Mb)^31–33^.

### Training and testing set

Features and corresponding MSI-PCR determined MSI status for all tumors were formulated into a large table (see Supplementary 1432_tumor_feature.csv). This table were then randomly split into two data sets, training and testing, according to a ratio of three to one for each tumor type, using a function called “train_test_split” provided by sklearn. Tables 1 and 2 shows the summary of these two datasets.

### Analysis of 22 features regarding their importance in MSI classification

We used the random forest algorithm to perform comprehensive analysis of all 22 features in order to gain their importance in MSI classification^23^. A random forest classifier was trained by the 1074-tumor training set using a random forest classifier framework provided by the python package scikit-learn (sklearn, 0.19.1)^30^. Importance scores of all features were then obtained from the “feature_importances_” attribute of the trained random forest classifier.

### Categorization of MSI

TCGA categorized MSI into three types using MSI-PCR: MSS (microsatellite stable), MSI-L (microsatellite instability low), and MSI-H (microsatellite instability high) in its clinical data. In this study, we grouped first two categories (MSS and MSI-L) together and denoted them as MSS because a recent study pointed out MSS and MSI-L tumors possess similar MSI burden^13^. We denoted MSI-H tumors as positive samples and MSS tumors as negative samples to make it more convenient for binary classification and model evaluation.

### Non-TCGA testing set

MAF files of 390 tumors, including 368 colorectal tumors and 22 stomach tumors generated by two non-TCGA projects^24,25^ together with their corresponding clinical data that embedded MSI-PCR determined binary (MSS and MSI-H) tumor MSI status, were directly downloaded from cBioPortal^26,27^. 22 features for mutational load characterization of each of these 390 tumors (see Supplementary non_tcga_390_tumor.csv) were extracted by MSIpred from their MAF files. Corresponding captured exome lengths (colorectal: 67 Mb, stomach: 38 Mb) required by MSIpred were obtained from their corresponding papers^24,25^. This data set was denoted as non-TCGA testing set, which was aimed to validate performance of MSIpred, and to conduct comparisons among original MSIpred (22 features), modified MSIpred (9 features), and MSIseq (10 features).

### Support vector machine

Support vector machines (SVMs) have been widely used in classification problems of computational biology due to high accuracy and flexibility in statistical modeling^36^. In this study, we utilized a SVM framework provided by the python package scikit-learn (sklearn, 0.19.1)^30^ to build our binary (MSS and MSI-H) classifier. As support vector machine is sensitive to scaling of features, our classifier actually was designed to incorporate a two-step framework: the first step is a standardized scaler provided by StandardScaler class of sklearn for feature normalization; the second step is a support vector machine classifier. A radial basis function (RBF) kernel of SVM was chosen and hyper parameters, gamma and C were tuned with 1074-tumor training data using grid search paired with ten-fold cross validation, which was achieved using GridSearchCV function of sklearn. The hyper parameter set yielding the largest average accuracy during cross validation was chosen. The SVM framework with optimized hyper-parameters was then trained with all training data (1074 tumors), and this trained SVM classifier was embedded in MSIpred.

### Model assessment metrics

As our 358-tumor testing set is imbalanced (280 MSS (negative) and 78 MSH(positive)), it is not sufficient to evaluate performance of our classifier by just using overall accuracy. Thus, we incorporated several other metrics for model evaluation. Equations of these metrics are given with following abbreviations: sensitivity (*Se*), specificity (*Sp*), precision (*Pre*), accuracy (ACC), FP (False Positive), FN (False Negative), TP (True Positive), TN (True Negative).

$$Se=\frac{TP}{TP+FN}$$

$$Sp=\frac{TN}{TN+FP}$$

$$Pre=\frac{TP}{TP+FP}$$

$$Acc=\frac{(TP+TN)}{(TP+TN+FP+FN)}$$

$$G\,Mean=\sqrt{Se\,\ast \,Sp}$$

$$F1\,Score=2\,\ast \,\frac{Pre\,\ast \,Se}{Pre+Se}$$

Receiver operating characteristic (ROC) curve and precision-recall curve were plotted to visualize the performance of MSIpred. Coordinate data used for plotting precision-recall curve and ROC curve was obtained by “precision_recall_curve” function and “roc_curve” function provided by sklearn, respectively. Visualizations were achieved using Matplotlib package.

### Comparisons among original MSIpred, modified MSIpred and MSIseq

We compared performances of our original MSIpred (22 features) with a modified version of MSIpred (9 features) and MSIseq (10 features) using the aforementioned 390-tumor non-TCGA testing dataset. The simple sequence repeats reference file required by these three tools was based on the same human reference genome (i.e., GRCh37) that the testing MAF files were based on. The modified MSIpred implemented a MSI classifier that was trained by just 9 (see Table S1 row 1–9) of 22 features of all tumors from the 1074-tumor training set. Performance comparisons of the original MSIpred, modified MSIpred and MSIseq were conducted by using aforementioned metrics.

### Statistical analysis

Wilcoxon rank sum tests were conducted using a built-in R (version 3.4.3) function wilcox.test. Data visualization was achieved using R package ggplot2 (version 2.2.1)^37^, python package Matplotlib (version 2.0.2)^38^, and python package Seaborn (version 0.8.0)^39^.

## Electronic supplementary material

Supplementary Information

1432_tumor_feature.csv

non_tcga_390_tumor.csv

## Data Availability

MSIpred is written in python 2. The package and detailed usage documents are freely available on GitHub: (https://github.com/bioinfolabmu/MSIpred). All the datasets generated and analyzed during the current study are available from the corresponding author on reasonable request.
