# Supplementary material for: MSIpred: a python package for tumor microsatellite instability classification from tumor mutation annotation data using a support vector machine
Source: Sci Rep. 2018 Dec 3;8:17546. doi: 10.1038/s41598-018-35682-z (PMC6277498; doi:10.1038/s41598-018-35682-z)
Supplement: Supplementary file 1 — Supplementary Information [file 41598_2018_35682_MOESM1_ESM.pdf]

MSIpred: a python package for tumor microsatellite instability classification from tumor mutation annotation data using a support vector machine

Chen Wang[1] and Chun Liang[1,2*]

[1]Department of Biology, Miami University, Oxford, OH 45056 USA
[2]Department of Computer Science & Software Engineering, Miami University, Oxford, OH 45056 USA

* Correspondence: liangc@miamioh.edu

Figure S1. Distributions of 22 features in 1432 tumors. A matrix of 22 histograms representing all features used by MSIpred. All 22 features display skewed distributions in 1432 tumors. Detailed definition for each feature can be found in supplementary Table S1. X axis of each subplot denotes value (counts per megabase) of each feature and Y axis denotes count of tumors.

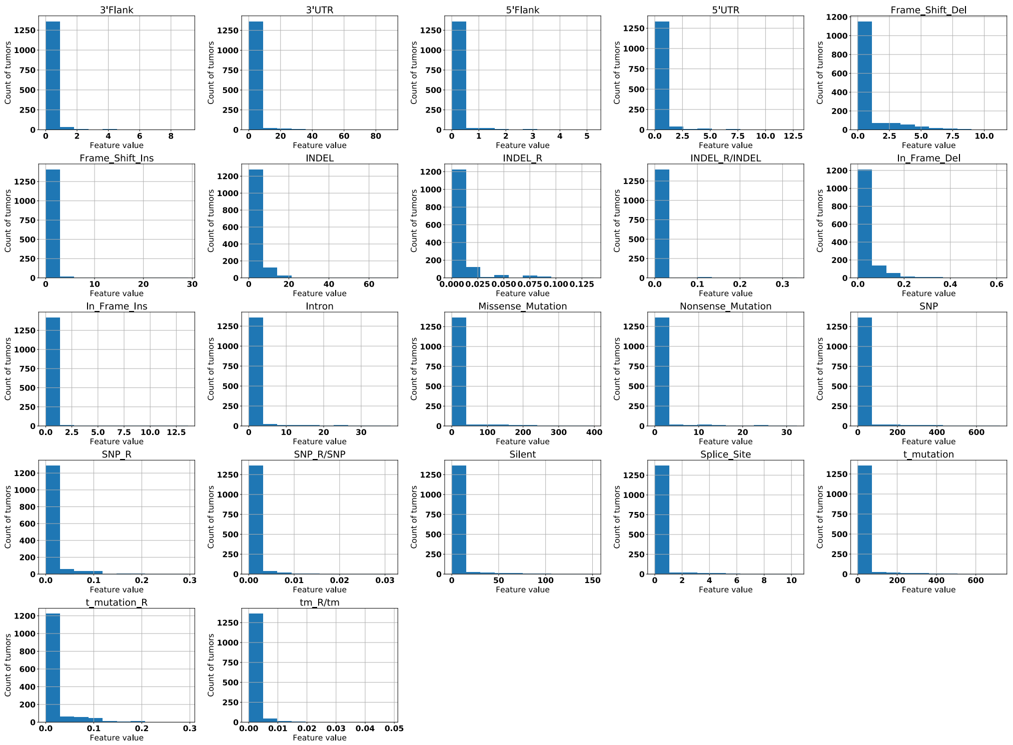

Figure S2. Pairwise scatter plots of 22 features used by MSIpred. A matrix of pairwise scatter plots of 22 features shows interactions between each pair of features. MSS tumors are labeled in blue while MSI-H tumors are labeled in orange. Histograms of all 22 features are placed on diagonal showing distributions of each feature in all 1432 tumors.

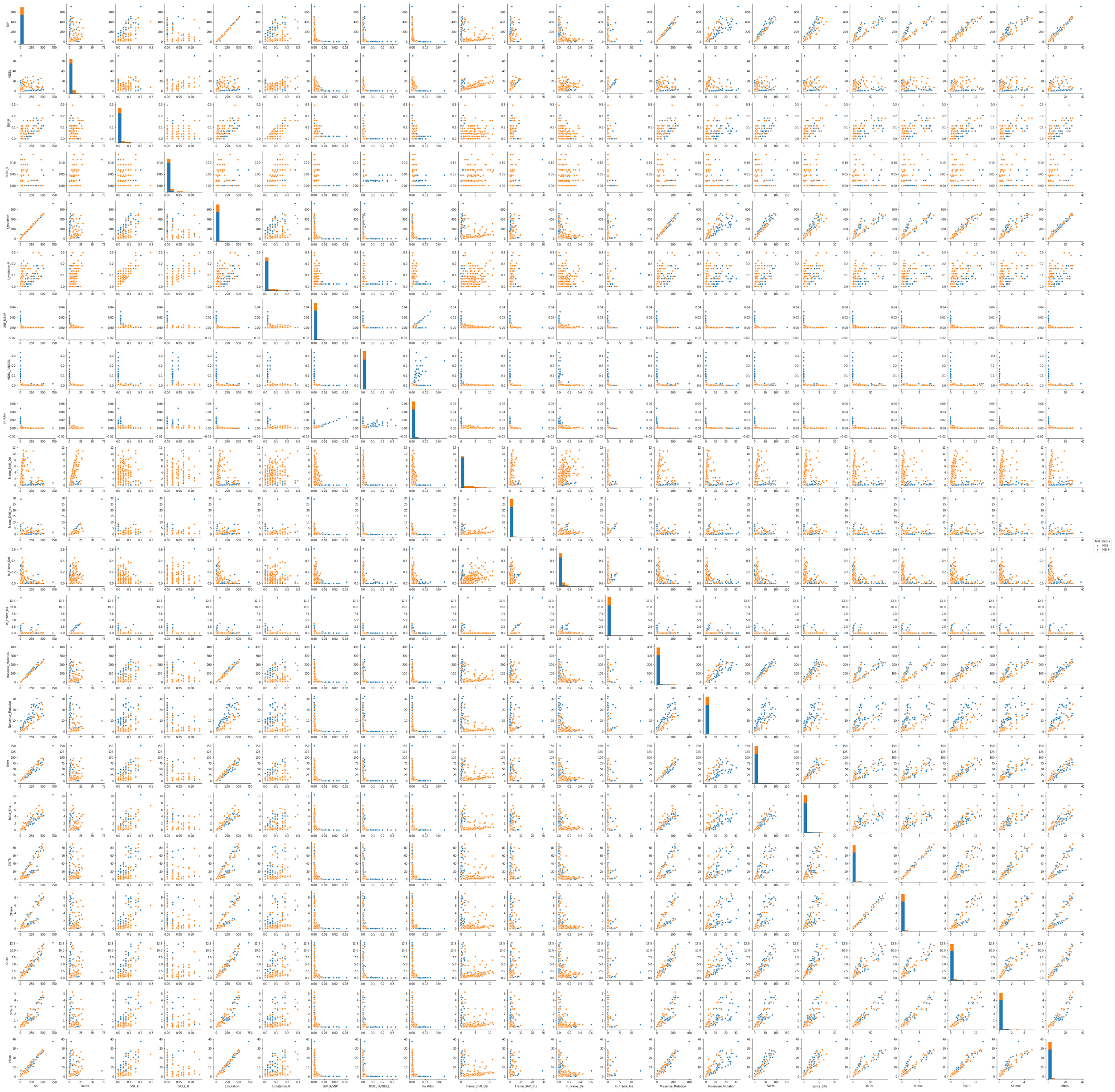

Table S1: Detailed definition of 22 features

No.	Features	Definition	Comment
**1**	SNP	counts of simple nucleotide substitution in all sequences per Mb	Variant Type
**2**	INDEL	counts of microindel in all sequences per Mb	Variant Type
**3**	SNP_R	counts of simple nucleotide substitution in simple repeat sequences per Mb	Variant Type
**4**	INDEL_R	counts of microindel in simple repeat sequences per Mb	Variant Type
**5**	t_mutation	total mutations (sum of SNP and INDEL) in all sequences per Mb	
**6**	t_mutation_R	total mutations (sum of SNP_R and INDEL_R) in simple repeat sequence per Mb	
**7**	SNP_R/SNP	Ratio of SNP_R over SNP	
**8**	INDEL_R/INDEL	Ratio of INDEL_R over INDEL	
**9**	tm_R/tm	Ratio of t_mutation_R over t_mutation	
**10**	Frame_Shift_Del	counts of deletion that cause shift of open reading frame per Mb	Variant Classification
**11**	Frame_Shift_Ins	counts of insertion that cause shift of open reading frame per Mb	Variant Classification
**12**	In_Frame_Del	counts of deletion that does not cause shift of open reading frame per Mb	Variant Classification
**13**	In_Frame_Ins	counts of insertion that does not cause shift of open reading frame per Mb	Variant Classification
**14**	Missense_Mutation	counts of missense mutation per Mb	Variant Classification
**15**	Nonsense_Mutation	counts of nonsense mutation per Mb	Variant Classification
**16**	Silent	counts of silent mutation per Mb	Variant Classification
**17**	Splice_Site	counts of mutation that locate at splice site per Mb	Variant Classification
**18**	3'UTR		Variant Classification

		counts of mutation that locate at 3'UTR region per Mb	
**19**	3'Flank	counts of mutation that locate at 3' Flank region per Mb	Variant Classification
**20**	5'UTR	counts of mutation that locate at 5'UTR region per Mb	Variant Classification
**21**	5'Flank	counts of mutation that locate at 5' Flank region per Mb	Variant Classification
**22**	Intron	counts of mutation that locate introns per Mb	Variant Classification

Table S2: Ranking of 22 features by their importance scores generated by training a random forest classifier with 1074-tumor training set

Feature	Importance
Frame_Shift_Del	0.4447
t_mutation	0.2876
Frame_Shift_Ins	0.0845
SNP	0.0843
INDEL	0.0154
5'Flank	0.0127
Missense_Mutation	0.0107
Splice_Site	0.0077
SNP_R/SNP	0.0072
In_Frame_Del	0.0068
Nonsense_Mutation	0.0064
3'UTR	0.006
In_Frame_Ins	0.0041
5'UTR	0.0037
Intron	0.0034
SNP_R	0.0029
tm_R/tm	0.0028
3'Flank	0.0028
t_mutation_R	0.0019
INDEL_R/INDEL	0.0018
Silent	0.0018
INDEL_R	0.001
